# Poor sleep is associated with energy drinks consumption among Korean adolescents

**DOI:** 10.1017/S136898002300191X

**Published:** 2023-12

**Authors:** Do Hee Kim, Bomgyeol Kim, Sang Gyu Lee, Tae Hyun Kim

**Affiliations:** 1 Department of Public Health, Graduate School, Yonsei University, Seodaemun-Gu, Seoul, Republic of Korea; 2 Department of Preventive Medicine, College of Medicine, Yonsei University, Seodaemun-Gu, Seoul, Republic of Korea; 3 Department of Healthcare Management, Graduate School of Public Health, Yonsei University, 50-1 Yonsei-Ro, Seodaemun-Gu, Seoul 03722, Republic of Korea

**Keywords:** Energy drinks, Sleep, Sleep duration, Sleep satisfaction, Adolescents

## Abstract

**Objective:**

A growing number of Korean adolescents consume energy drinks, which may increase the risk of obesity, anxiety and insomnia. We examined whether poor sleep was associated with energy drink consumption among study participants.

**Design:**

We used a cross-sectional design.

**Setting:**

The Korea Youth Risk Behavior Web-Based Survey data from 2019.

**Participants:**

To determine the association between sleep and energy drink consumption, we compared the independent variables for 50,455 adolescents in Korea (aged 14–19 years) using multivariate logistic regression and sensitivity analyses.

**Results:**

In Korea, 69·5 % adolescents consumed energy drinks, 17·1 % slept for less than 5 h, 22·4 % slept for 5–6 h, 23·8 % slept for 6–7 h, 19·9 % slept for 7–8 h and 16·7 % slept for 8 h or more. Regarding sleep satisfaction, 21·0 % reported sufficient, 32·6 % reported just enough and 46·5 % reported insufficient. Regarding sleep duration, it was found that less than 5 h (OR, 2·36; 95 % CI (2·14, 2·60)) and lower sleep satisfaction (OR, 1·12; 95 % CI (1·03, 1·21)) were highly associated with energy drink consumption, with statistical significance at *P* < 0·05. Adolescents with lower sleep duration (adjusted OR (aOR), 6·37; 95 % CI (4·72, 8·61)) and a lack of sleep satisfaction (aOR, 1·44; 95 % CI (1·16, 1·78)) reported drinking a high amount of energy drinks, that is, at least once a day.

**Conclusion:**

In addition to efforts to decrease the amount of energy drinks consumed, sleep hygiene education needs to be strengthened.

Sleep is essential for the optimal health of children and adolescents^(1)^. In 2015, a study recommended that adolescents (aged 14–17 years) must have a minimum of 8–10 h of sleep a night^(2)^. The nightly sleep duration of adolescents in Korea is 4·9–6·5 h of sleep, which is less than the recommended sleep duration^(3)^.

Poor sleep is associated with disturbances in cognitive and psychomotor functions, including mood, thinking, concentration, memory, learning, vigilance and reaction time^(4–6)^. Chronic sleep deprivation poses a serious threat to the academic success, health and safety of adolescents^(7)^. Poor sleep in adolescence is associated with negative outcomes in several areas of health and functioning, including obesity, depression, school performance, quality of life and risk-taking behaviours^(8)^. Poor sleep is caused by inconsistent class schedules that vary from day to day, early or late obligations, use of technology and stimulants, and consumption of caffeine and energy drinks^(9)^.

In particular, the consumption of energy drinks increases sleep latency, the effects of which can persist for up to 8 h^(9)^. Energy drinks contain high levels of caffeine in combination with ingredients such as amino acids, sugars, sweeteners, guarana, taurine, ginseng, L-carnitine, herbal supplements and vitamin B^(10)^. Energy drink consumption has become increasingly widespread among adolescents owing to targeted marketing and the marked effects of providing a quick boost in energy, increasing alertness and ease of availability^(11)^.

An American study that analysed data from 2003 to 2016 found that energy drink consumption significantly increased among adolescents over that period and that boys consumed energy drinks at a significantly higher level than girls across all age groups^(12)^. According to previous studies, energy drinks pose several challenges. First, they are associated with an increase in heart rate and arterial blood pressure. Second, high-energy drink consumption may increase the risk of obesity and type 2 diabetes mellitus. Third, energy drink consumption leads to symptoms of anxiety, insomnia, gastrointestinal upset, muscle twitching, restlessness and periods of inexhaustibility^(13,14)^.

Despite their harmful effects, as energy drinks counteract the effects of insufficient sleep, adolescents consume excessive amounts of these drinks^(15,16)^. In Korea, adolescent energy drink consumption increased from 2015 to 2020^(17)^. The reasons for energy drink consumption were revealed to be taste, followed by the energy boost provided by these drinks. Awakeness has also been reported as a reason^(18)^. Adolescents who sleep late consume more energy drinks to stay awake^(19)^.

Almost previous studies have reported that consumption of energy drinks is associated with poor sleep^(20,21)^. While other studies have investigated the association between sleep duration and energy drink consumption or sleep satisfaction and energy drink consumption, our research stands out in that it examines the association between sleep and energy drink consumption using both of sleep duration and sleep satisfaction^(22,23)^. Thus, we hypothesise that poor sleep is associated with higher amount of energy drinks consumption.

This study investigated the association between sleep (sleep duration and sleep satisfaction) and energy drink consumption in adolescents using representative data from the 15th Korea Youth Risk Behavior Web-Based Survey (KYRBS) of Korean adolescents.

## Methods

### Study population and data collection

This study used data from the 15th KYRBS conducted in 2019 by the Korea Disease Control and Prevention Agency (KDCA). The KYRBS is an ongoing national cross-sectional, self-administered, structured questionnaire with a complex research design that includes multistage cluster sampling. The purpose of the KYRBS is to identify health behaviours under the present conditions, report health indicators to plan and appraise health promotion for adolescents, and compare the health indicators of adolescents from different countries. The target participants consisted of middle and high school students from seventeen Korean provinces, including public and private institutions. The stratified cluster random sampling method was used for sampling; individual schools were chosen as the primary sample units (PSU) and selected as the permanent random sample for each cluster. The classroom was considered the secondary sample, and one classroom was randomly extracted for each grade from the selected sample school.

KYRBS uses an online survey system that does not allow respondents to proceed to the next section of the questionnaire unless all questions in the current section are answered. Responses with logical errors or outliers are processed as missing values. The questionnaire contained approximately 120 items across fifteen categories, including demographic characteristics and health-associated behaviours. Middle school students (aged 14–16 years) and high school students (aged 17–19 years) were the target populations in our study. This survey included 57 303 adolescents, of which 50 455 participants were selected for the final analysis after excluding those with missing data.

Ethics approval for KYRBS was waived by the KDCA’s Institutional Review Board in accordance with the Bioethics and Safety Act of 2015. All KYRBS participants provided informed consent. KYRBS fully complied with the rules outlined by the Declaration of Helsinki.

### Measurements

#### Dependent variable

Energy drink consumption was the dependent variable. To measure the same, the question ‘During the last 7 d, how often have you consumed energy drinks?’ was scored on the following scale: haven’t drunk in the last 7 d, 1–2 times a week, 3–4 times a week, 5–6 times a week, 1 time a day, 2 times a day and more than 3 times a day. In this study, participants who did not drink in the last 7 d were reclassified as ‘no’ and the others were considered ‘yes’.

#### Independent variables

##### Variables of interest

Sleep characteristics included sleep duration and satisfaction. Sleep duration was answered by the question ‘During the last 7 d, what time did you usually wake up and go to bed?’ for the following two periods: weekdays (Monday to Friday) and weekends (Saturday to Sunday). In this study, sleep duration was calculated by subtracting bedtime on weekdays from waking time and was divided into five categories: less than 5 h, 5–6 h, 6–7 h, 7–8 h, and 8 h or more. Sleep satisfaction was answered by the question ‘During the last 7 d, how satisfied have you felt with your hours of sleep in terms of fatigue recovery?’ The answers were very sufficient, sufficient, just enough, not enough or not enough at all. In this study, we reclassified sufficient and sufficient as ‘sufficient’, just enough as ‘just enough’, and not enough and not enough at all as ‘not enough’.

##### Covariates

Factors related to energy drink consumption were classified into general and health-related characteristics. General characteristics included the sex, school year, subjective academic performance and subjective economic status. The school year was divided into ‘middle school (14–16 years old)’ and ‘high school (17–19 years old)’. Subjective academic performance was scored based on academic performance in the last 12 months as high, mid-high, medium, mid-low or low. Earlier studies on health behaviour and perceived stress of high school students were classified by reference^(18)^. The detailed categories were reclassified as ‘medium and above’ and ‘mid-low and lower’, where high, mid-high, and medium were categorised as ‘medium and above’, and mid-low and low were categorised as ‘mid-low and lower’. Subjective economic status was rated as high, mid-high, medium, mid-low or low. In this study, high and mid-high were reclassified as ‘high’, medium as ‘medium’, and mid-low and low as ‘low’.

Health-related characteristics included subjective health status, perceived stress, BMI, asthma history and physical activity. Subjective health status was determined by the question ‘How do you usually rate your health?’ Answers such as ‘very healthy’ and ‘healthy’ were classified as ‘good’, ‘normal’ as ‘normal’, and ‘unhealthy’ and ‘very unhealthy’ as ‘poor’. The level of perceived stress was categorised as: ‘very much’, ‘much’, and ‘a little’ as ‘more stressed’ and ‘not much’ and ‘not at all’ as ‘less stressed’. According to the 2017 Korean Pediatric Society’s standard growth charts for Korean children and adolescents, four categories were created: underweight (≤ 15th quartile), normal weight (16th–84th quartile), overweight (85th–95th quartile) and obesity (≥ 95th quartile or BMI ≥ 25)^(24)^. Asthma history was defined by answering ‘yes’ to the following question: ‘Have you been diagnosed with asthma by a doctor?’ Physical activity was defined by answering the question ‘During the last 7 d, on how many days did your heart rate increase from normal, or did you have a total of 60 min or more per day (regardless of type) of more than normal physical activity?’ The answers were not in the last 7 d, 1 d a week, 2 d a week, 3 d a week, 4 d a week, 5 d a week, 6 d a week or every day. In this study, not in the last 7 d was reclassified as ‘not in the last 7 d’, 1 d a week, 2 d a week, and 3 d a week as ‘1–3 times a week’, 4 d a week and 5 d a week as ‘4–5 times a week’, and 6 d a week and every day as ‘6–7 times a week’.

### Statistical analyses

KYRBS data were surveyed via systematic sampling and contained weighted values; hence, statistical analysis was performed by applying weighted values^(25)^. Independent variables were compared using the chi-square test to determine their association with energy drink consumption. We performed multivariate logistic regression analysis to evaluate the association between poor sleep and higher amount of energy drink consumption as one model for all categories, and the results were reported as adjusted OR (aOR) and 95 % CI. Furthermore, sensitivity analysis, that is, multinomial logistic regression analysis^(26)^, was conducted to investigate the association between sleep and consumption amount of the energy drinks after classifying by adjusting the selected covariates. The differences were considered statistically significant level of *P*-value (*P* < 0·05). All statistical analyses were performed using SAS version 9.4 (SAS Institute Inc.).

## Results

### Participant characteristics

In total, 50 445 adolescents from the original sample were included in this study. Participants’ characteristics are presented in Table 1. Sleep pattern characteristics: the majority of respondents are reported sleeping duration about 6–7 h (23·8 %), followed by 5–6 h (22·4 %), 7–8 h (19·9 %), less than 5 h (17·1 %) and more than 8 h (16·7 %). While the recommended sleep duration for adolescents is more than 8 h, the recommended sleep duration satisfaction rate for Korean adolescents was only 16·7 %. Over the past 7 d, almost half of the adolescents, 46·5 %, reported insufficient sleep satisfaction. This was followed by just enough sleep satisfaction reported by 32·6 % and sufficient sleep satisfaction reported by 21·0 %. Regarding energy drink consumption, 30·4 % Korean adolescents reported that they consumed energy drinks more than once a week.

**Table 1:** Summary statistics (*n* 50 455)

Variables	Categories	*n*	%
Energy drink consumption	No	35 295	69·6
	Yes	15 150	30·4
Sleep duration	Less than 5 h	8123	17·1
	5–6 h	10 814	22·4
	6–7 h	11 918	23·8
	7–8 h	10 454	19·9
	8 h or more	9136	16·7
Sleep satisfaction	Sufficient	10 913	21·0
	Just enough	16 512	32·6
	Not enough	23 020	46·5
Sex	Boys	26 014	51·5
	Girls	24 331	48·5
School year	Middle school (14–16 years old)	25 685	47·6
	High school (17–19 years old)	24 760	52·4
Subjective academic performance	Medium and above	15 603	31·1
	Mid–low and lower	34 842	68·9
Subjective economic status	High	19 790	39·7
	Middle	24 337	48·1
	Low	6318	12·3
Subjective health status	Good	35 752	70·6
	Normal	11 212	22·4
	Bad	3481	7·0
Perceived stress	Less stressed	9769	19·1
	More stressed	40 676	80·9
BMI (kg/m^2^)	Normal weight	36 403	7·1
	Underweight	3518	72·3
	Overweight and obesity	10 524	20·6
Asthma history	No	47 012	93·0
	Yes	3433	7·0
Physical activity*	0	17 576	35·4
	1–3	21 822	43·4
	4–5	6647	12·9
	6–7	4400	8·3

* Days when the heart rate increases from normal or is more than 60 min total per d with more physical activity than normal (regardless of type).

In general, most of the participants were boys (51·5 %) and high school students (52·4 %). They had mid-low or lower subjective academic performance (68·9 %) and a medium subjective economic status (48·1 %). Regarding health-related behaviours, most of the participants had a good subjective health status (70·6 %), perceived more stress (80·9 %), underweight (72·3 %), no experience of asthma (93·0 %) and reported 1–3 d a week of physical activity (43·4 %) (Table 1).

### Differences in energy drink consumption according to participant characteristics

Regarding the relationship between sleep characteristics and energy drink consumption, energy drink consumption was reported by 42·0 % of those with less than 5 h of sleep, 33·2 % of those with 5–6 h, 29·4 % with 6–7 h, 24·9 % with 7–8 h and 22·5 % with 8 h or more (*P* < 0·001). Regarding sleep satisfaction among those who reported consuming energy drinks, 25·1 % were satisfied, 28·9 % said they had just enough sleep and 33·8 % reported insufficient sleep and consuming energy drinks (*P* < 0·001).

Regarding general characteristics, 32·1 % of boys and 28·6 % of girls reported energy drink consumption (*P* < 0·001); by school year, 26·6 % were in middle school (14–16 years old) and 33·8 % were in high school (17–19 years old); and by subjective academic performance, 29·5 % had mid-low and lower performance records and 32·3 % had mid-level and higher records (*P* < 0·001). Subjective economic status was high in 30·9 %, medium in 29·6 % and low in 32·8 % of participants (*P* = 0·0013).

Regarding health-related behaviours, among adolescents consuming energy drink was reported as follows: based on subjective health status, 29·3 % reported good, 30·4 % reported normal and 35·0 % reported bad (*P* < 0·001); based on perceived stress, 31·7 % reported being more stressed and 24·9 % reported being less stressed (*P* < 0·001); by BMI, 29·9 % were normal weight, 28·4 % were underweight and 32·7 % were overweight or obese (*P* < 0·001); as for asthma history, 30·2 % were in the ‘No’ group and 33·2 % were in the ‘Yes’ group (*P* = 0·0008). Regarding physical activity, energy drink consumption was reported as follows: 28·2 % reported ‘not in the last 7 d’, 31·4 % reported ‘1–3 d a week’, 30·9 % reported ‘4–5 d a week’ and 33·4 % reported ‘6–7 d a week’ (*P* < 0·001; Table 2).

**Table 2:** General characteristics of the participants by energy drinks consumption

Variables	Categories	Energy drink consumption				*P*-value
		No		Yes		
		*n*	%	*n*	%	
Sleep duration	Less than 5 h	4736	58·0	3387	42·0	<0·0001
	5–6 h	7256	66·8	3558	33·2	
	6–7 h	8449	70·6	3469	29·4	
	7–8 h	7853	75·1	2601	24·9	
	8 h or more	7001	77·5	2135	22·5	
Sleep satisfaction	Sufficient	8163	74·9	2750	25·1	<0·0001
	Just enough	11 749	71·1	4763	28·9	
	Not enough	15 383	66·2	7637	33·8	
Sex	Boys	17 768	67·9	8246	32·1	<0·0001
	Girls	17 527	71·4	6904	28·6	
School year	Middle school (14–16 years old)	18 830	73·4	6855	26·6	<0·0001
	High school (17–19 years old)	16 465	66·2	8295	33·8	
Subjective academic performance	Medium and above	24 715	70·5	10 127	29·5	<0·0001
	Mid-low and lower	10 580	67·7	5023	32·3	
Subjective economic status	High	13 727	69·1	6063	30·9	0·0013
	Middle	17 225	70·4	7112	29·6	
	Low	4343	68·2	1975	31·8	
Subjective health status	Good	25 390	70·7	10 362	29·3	<0·0001
	Normal	7633	69·6	3579	30·4	
	Bad	2272	65·0	1209	35·0	
Perceived stress	Less stressed	7351	75·1	2418	24·9	<0·0001
	More stressed	27 944	68·3	12 732	31·7	
BMI (kg/m^2^)	Normal weight	25 597	70·1	10 806	29·9	<0·0001
	Underweight	2529	71·6	989	28·4	
	Overweight and obesity	7169	67·3	3355	32·7	
Asthma history	No	32 991	69·8	14 021	30·2	0·0008
	Yes	2304	66·8	1129	33·2	
Physical activity	0	12 719	71·8	4857	28·2	<0·0001
	1–3	14 995	68·6	6827	31·4	
	4–5	4638	69·1	2009	30·9	
	6–7	2943	66·6	1457	33·4	

Values are presented as numbers (%).

### Relationship between participant characteristics and energy drink consumption

Regarding sleep duration characteristics, shorter sleep duration was related to energy drink consumption. It was found that adolescents with fewer than 5 h of sleep (OR, 2·36; 95 % CI (2·14, 2·60)) consumed more energy drinks than adolescents with 7–8 h of sleep (OR, 1·12; 95 % CI (1·03, 1·23)), 6–7 h of sleep (OR, 1·38; 95 % CI (1·26, 1·51)) and 5–6 h of sleep (OR, 1·62; 95 % CI (1·48, 1·78)). Those with lower sleep satisfaction consumed more energy drinks than those with sufficient sleep satisfaction (aOR, 1·07; 95 % CI (1·01, 1·14) for just enough and aOR, 1·14; 95 % CI (1·07, 1·21) for not enough). Therefore, sleep (sleep duration and sleep satisfaction) has a negative relationship with energy drink consumption.

The sex-based energy drink consumption showed that boys had higher consumption than girls (aOR, 0·76; 95 % CI (0·72, 0·80)), whereas high school students (aOR, 1·07; 95 % CI (1·01, 1·14)) consumed energy drinks more often than middle school students. Regarding subjective academic performance (grades), we found that mid-low and lower-grade students (aOR, 1·12; 95 % CI (1·06, 1·18)) and those with a high economic status (aOR, 1·10; 95 % CI (1·03, 1·19)) consumed energy drinks more than their selected reference group. However, there was no difference between middle subjective economic status and energy drink consumption (aOR, 0·99; 95 % CI (0·93, 1·06)).

For subjective health status, energy drink consumption was higher for those with poorer health (aOR, 1·11; 95 % CI (1·05, 1·17) for normal health; aOR, 1·14; 95 % CI (1·05, 1·23) for poor health). The proportion of respondents who were more stressed and reported energy drink consumption was higher than those who were less stressed (aOR, 1·25; 95 % CI (1·18, 1·33)). For BMI, the proportions of those who were overweight and obese who reported energy drink consumption were higher than those who belonged to the normal weight category (aOR, 1·07; 95 % CI (1·02, 1·13)). However, there was no difference between being underweight and energy drink consumption (aOR, 0·94; 95 % CI (0·86, 1·03)). In addition, we did not observe any difference between asthma history and energy drink consumption. Finally, for physical activity, the proportion reporting energy drink consumption among those who engaged in physical activity was higher than that among those who did not engage in physical activity (aOR, 1·18; 95 % CI (1·12, 1·24) for 1–3 times a week; aOR, 1·20; 95 % CI (1·11, 1·29) for 4–5 times a week; aOR, 1·35; 95 % CI (1·24, 1·47) for 6–7 times a week) (Table 3).

**Table 3:** Logistic regression on energy drink consumption

Variables	Categories	aOR	95 % CI	*P*
Sleep duration	8 h or more	Ref.		
	7–8 h	1·12	1·03, 1·23	0·0079
	6–7 h	1·38	1·26, 1·51	<0·0001
	5–6 h	1·62	1·48, 1·78	<0·0001
	Less than 5 h	2·36	2·14, 2·60	<0·0001
Sleep satisfaction	Sufficient	Ref.		
	Just enough	1·07	1·01, 1·14	0·0261
	Not enough	1·14	1·07, 1·21	0·0001
Sex	Boys	Ref.		
	Girls	0·76	0·72, 0·80	<0·0001
School year	Middle school (14–16 years old)	Ref.		
	High school (17–19 years old)	1·07	1·01, 1·14	0·0321
Subjective academic performance	Medium and above	Ref.		
	Mid-low and lower	1·12	1·06, 1·18	<0·0001
Subjective economic status	Low	Ref.		
	Middle	0·99	0·93, 1·06	0·7701
	High	1·10	1·03, 1·19	0·0068
Subjective health status	Good	Ref.		
	Normal	1·11	1·05, 1·17	0·0001
	Bad	1·14	1·05, 1·23	0·0023
Perceived stress	Less stressed	Ref.		
	More stressed	1·25	1·18, 1·33	<0·0001
BMI (kg/m^2^)	Normal weight	Ref.		
	Underweight	0·94	0·86, 1·03	0·1741
	Overweight and obesity	1·07	1·02, 1·13	0·0119
Asthma history	No	Ref.		
	Yes	1·07	0·98, 1·16	0·1148
Physical activity	0	Ref.		
	1–3	1·18	1·12, 1·24	<0·0001
	4–5	1·20	1·11, 1·29	<0·0001
	6–7	1·35	1·24, 1·47	<0·0001

### Relationship between characteristics of sleep and amount of energy drink consumption

**Fig. 1:**
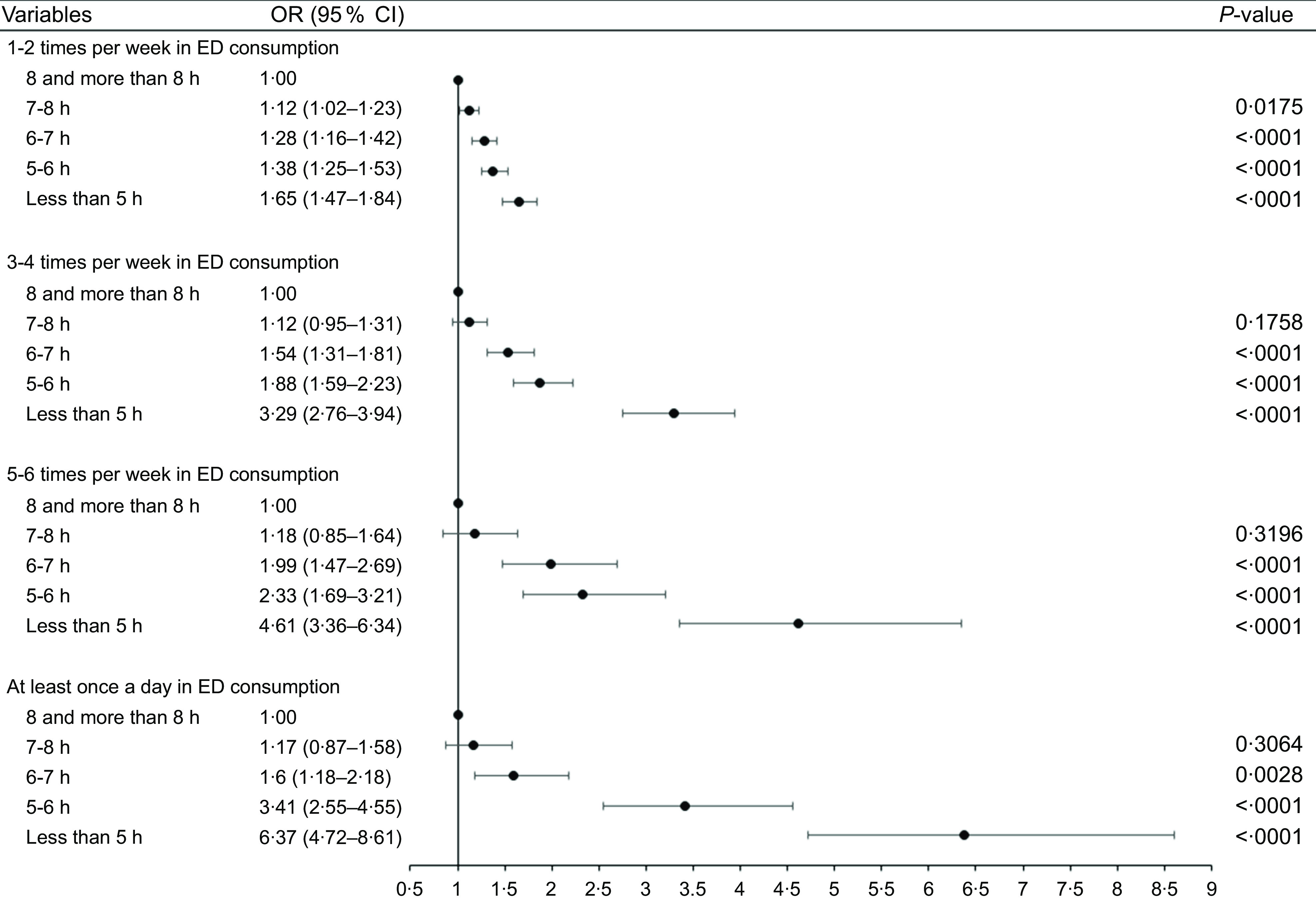
Result of sensitivity analysis of multinomial logistic regression between sleep duration and amount of energy drink (ED) consumption

Table 4 and Figure 1 illustrate the multinomial logistic regression results of the sensitivity analysis. The relationship between sleep duration and amount of energy drink consumption showed that adolescents with less than 5 h of sleep (aOR, 6·37; 95 % CI (4·72, 8·61)) had the highest relationship with energy drink consumption (i.e. at least once a day), followed by 5–6 times per week (aOR, 4·61; 95 % CI (3·36, 6·34)) and 3–4 times per week (aOR, 3·29; 95 % CI (2·76, 3·94)). The results showed a significant difference (*P* < 0·05). In terms of sleep satisfaction, the adolescents who had not enough sleep satisfaction (aOR, 1·44, 95 % CI (1·16, 1·78)) were likely to consume more drinks about 5–6 times per week. However, except for at least once per d (*P* = 0·489), the other categories showed partially significant differences.

**Table 4:** Sensitivity analysis: multinomial analysis of the association between sleep and energy drink consumption

Variables	Categories	1–2 times/week			3–4 times/week			5–6 times/week			At least once a day		
		aOR	95 % CI	*P*	aOR	95 % CI	*P*	aOR	95 % CI	*P*	aOR	95 % CI	*P*
Sleep duration	8 h or more	Ref.			Ref.			Ref.			Ref.		
	7–8 h	1·12	1·02, 1·23	0·0175	1·12	0·95, 1·31	0·1758	1·18	0·85, 1·64	0·3196	1·17	0·87, 1·58	0·3064
	6–7 h	1·28	1·16, 1·42	<0·0001	1·54	1·31, 1·81	<0·0001	1·99	1·47, 2·69	<0·0001	1·60	1·18, 2·18	0·0028
	5–6 h	1·38	1·25, 1·53	<0·0001	1·88	1·59, 2·23	<0·0001	2·33	1·69, 3·21	<0·0001	3·41	2·55, 4·55	<0·0001
	Less than 5 h	1·65	1·47, 1·84	<0·0001	3·29	2·76, 3·94	<0·0001	4·61	3·36, 6·34	<0·0001	6·37	4·72, 8·61	<0·0001
Sleep satisfaction	Sufficient	Ref.			Ref.			Ref.			Ref.		
	Just enough	1·12	1·05, 1·21	0·0017	0·99	0·88, 1·12	0·9071	1·24	0·99, 1·56	0·0607	0·74	0·61, 0·91	0·0032
	Not enough	1·11	1·03, 1·21	0·0079	1·13	1·01, 1·27	0·0361	1·44	1·16, 1·78	0·0009	1·07	0·88, 1·3	0·4895

aOR, adjusted OR.

## Discussion

This study used a large sample of 2019 KYRBS data from a national survey based on a sample of Korean adolescents. As hypothesised, poor sleep was associated with a higher amount of consumption energy drink consumption. Our results showed that adolescents who did not sleep long enough or had lower sleep satisfaction consumed energy drinks at higher frequencies. In addition, our study found associations between energy drink consumption and boys, high school students, high subjective academic status, bad subjective health status, more perceived stress, and more physical activity. However, mixed results were found for associations between energy drink consumption and subjective economic status and BMI.

Our findings are consistent with those of previous studies^(23,24,27,28)^. Sampasa-Kanyinga *et al.* found that shorter sleep duration was associated with higher energy drink consumption^(29)^. Lohsoonthorn *et al*. found that lower sleep satisfaction was related to energy drink consumption in adolescents^(30)^. However, previous studies have reported that the consumption of energy drinks is associated with poor sleep, which can impact the onset of sleep and reduce sleep time, efficiency, and satisfaction levels^(20,21)^. Conversely, we found that poor sleep was associated with the consumption of energy drinks. Several studies have investigated the association between sleep and energy drink consumption, but this study was the first to examine the association between sleep (sleep duration and sleep satisfaction) and energy drink consumption^(22,23,28)^. Moreover, this study adds to the growing body of literature on the relationship between sleep and unhealthy beverages. For example, adolescents who slept for a short duration and had low satisfaction with this level of sleep had higher odds of consuming sugar-sweetened beverages^(31,32)^. To the best of our knowledge, this is the first study in Korea to collectively investigate sleep duration and sleep satisfaction to determine their association with energy drink consumption.

Furthermore, the covariates were consistent with those of previous studies. Boys were more likely to consume energy drinks than girls, and high school students were more likely to consume energy drinks than middle school students. Our results are consistent with the finding that the consumption of energy drinks more than once a week was more frequent among boys than girls^(15,16,19)^, which is consistent with previous studies showing that high school students consume more energy drinks than middle school students^(22,25,33)^. Adolescents who engaged in physical activity in our study consumed more energy drinks than those who did not. Park *et al*. determined that the consumption of energy drinks was higher among those who engaged in physical activity 7 or more times a week compared to those who did so twice a week or less^(34)^. Some studies have found that physical activity is associated with improved sleep quality^(26–29)^ and higher energy drink consumption^(20–24)^. However, we observed no evidence of an association between overall sleep quality and physical activity in our study. Instead, the more stressed groups and those with mid-low or lower academic performance records consumed more energy drinks.

Many prior studies have suggested that adolescents consume energy drinks to stay awake^(19,35)^. Korean adolescents’ academic performance is vital for their psychosocial development and preparation to enter college and adulthood. High school students in Korea attend classes between 08:00 and 17:00. Nearly, all Korean high school students attend private after-school institutions or stay at school for evening studies that may continue until 22:00 h^(36)^. Under the strong sociocultural and psychological influence of college entrance examinations, most Korean adolescents further curtail their sleep duration^(37)^. Sleep dissatisfaction causes tiredness/fatigue^(13)^. Furthermore, a previous study showed that shorter sleep duration is related to daytime sleepiness and poor daytime functioning^(7)^. Consequently, daytime sleepiness occurs owing to a lack of sleep, which leads to energy drink consumption to stay awake^(38)^. However, our results showed that the mid-low and lower academic groups consumed more energy drinks than the medium and above groups. In fact, people believe that consuming energy drinks helps them with their homework and study^(39)^. Many students currently believe that using energy drinks will facilitate their study and work on school projects^(40)^. However, the effects of high caffeine consumption on stress and sleep can lead to inefficient learning^(36,41)^.

It would be better for adolescents to get sufficient sleep; however, this may not be feasible because of their many classes and studies^(42)^. Poor sleep and energy drink consumption are not beneficial to adolescent health, and public health attention and management are needed. Sleep hygiene education improves sleep quality by providing comprehensive education regarding the harmful effects of caffeine, tobacco and alcohol. It encourages adolescents to build exercise routines, reduce stress, and manage their sleep and napping times^(2)^. Thus, sleep hygiene education may be considered a method to address the issue of energy drink consumption.

### Limitations

This study has some limitations. First, although results showed the association that poor sleep was associated with higher amount of energy drinks consumption, the data were obtained from a cross-sectional survey; thus, this study can only indicate statistical associations and has a problem of reverse causality. Second, the data were based on self-reported data provided by the respondents online. The survey respondents may have understood or overstated their responses. Third, despite the significant emotional understanding of Korean characteristics of increasing adolescents’ anxiety under the strong sociocultural and psychological influence of college entrance examinations, there were data limitations such as the lack of test anxiety in the variables. Therefore, future studies should address the association between sleep and energy drink consumption, including psychological variables such as academic anxiety and stress caused by academic demands. Fourth, this study used data from 2019, which may have affected its interpretation. The data may have been amplified or attenuated during the pandemic period.

## Conclusion

In conclusion, this study showed that poor sleep is associated with higher amount of energy drinks consumption among Korean adolescents. As the number of adolescents who consume energy drinks is increasing, efforts should be made to provide sleep hygiene education to enable adolescents to better understand sleep hygiene, improve sleep duration and satisfaction, and reduce the consumption of energy drinks.
